# Longitudinal analysis of direct and indirect effects on average daily gain in rabbits using a structured antedependence model

**DOI:** 10.1186/s12711-018-0395-9

**Published:** 2018-05-10

**Authors:** Ingrid David, Juan-Pablo Sánchez, Miriam Piles

**Affiliations:** 1 GenPhySE, INRA, Université de Toulouse, INPT, ENVT, 31326 Castanet Tolosan, France; 2 Institute for Food and Agriculture Research and Technology, Torre Marimon s/n, 08140 Caldes de Montbui, Barcelona Spain

## Abstract

**Background:**

Indirect genetic effects (IGE) are important components of various traits in several species. Although the intensity of social interactions between partners likely vary over time, very few genetic studies have investigated how IGE vary over time for traits under selection in livestock species. To overcome this issue, our aim was: (1) to analyze longitudinal records of average daily gain (ADG) in rabbits subjected to a 5-week period of feed restriction using a structured antedependence (SAD) model that includes IGE and (2) to evaluate, by simulation, the response to selection when IGE are present and genetic evaluation is based on a SAD model that includes IGE or not.

**Results:**

The direct genetic variance for ADG (g/d) increased from week 1 to 3 [from 8.03 to 13.47 (g/d)^2^] and then decreased [6.20 (g/d)^2^ at week 5], while the indirect genetic variance decreased from week 1 to 4 [from 0.43 to 0.22 (g/d)^2^]. The correlation between the direct genetic effects of different weeks was moderate to high (ranging from 0.46 to 0.86) and tended to decrease with time interval between measurements. The same trend was observed for IGE for weeks 2 to 5 (correlations ranging from 0.62 to 0.91). Estimates of the correlation between IGE of week 1 and IGE of the other weeks did not follow the same pattern and correlations were lower. Estimates of correlations between direct and indirect effects were negative at all times. After seven generations of simulated selection, the increase in ADG from selection on EBV from a SAD model that included IGE was higher (~ 30%) than when those effects were omitted.

**Conclusions:**

Indirect genetic effects are larger just after mixing animals at weaning than later in the fattening period, probably because of the establishment of social hierarchy that is generally observed at that time. Accounting for IGE in the selection criterion maximizes genetic progress.

**Electronic supplementary material:**

The online version of this article (10.1186/s12711-018-0395-9) contains supplementary material, which is available to authorized users.

## Background

The genes that an individual carries and the environment in which it lives determine its phenotype. In various situations, during the pre-weaning period or in the case of group housing for instance, other individuals are part of the environment of the focus animal. Thus, the phenotype of an individual is influenced by its interactions with other individuals of the group. Such interactions may be positive (e.g. maternal effects or sterile helpers in social insects [1]) or negative (e.g. competition for limited resources or aggressive social behavior [2–4]) and are driven by other phenotypes of the group mates that generally are not recorded or measurable but can have a genetic component. Consequently, genetics of group mates or the dam may influence the focal phenotype. Such effects are known as indirect genetic effects (IGE) [5, 6] and have been reported to be important components of various traits in different species [2–4, 7]. Their role in the evolutionary processes of wild species and in response to selection in livestock species have also been explored [8].

The intensity of social interactions can depend highly on external factors, such as the number of interacting individuals [9, 10] and rearing conditions. For instance, Piles et al. [7] recently showed that IGE for average daily gain (ADG) were stronger for rabbits under a restricted feeding regime than for rabbits fed ad libitum. In addition, interactions between group members can also vary over time. Several studies have shown that aggressive behavior of animals in group housing conditions is generally stronger at mixing and tends to decrease over time [11–13], which suggests that IGE can vary over time. Nonetheless, very few genetic studies have investigated changes of IGE over time for traits under selection in livestock species. To overcome this, the objectives of our study were to analyze longitudinal records of ADG in rabbits using a structured antedependence (SAD) model [14] that includes IGE and to evaluate, by simulation, response to selection on longitudinal ADG using different selection strategies. The idea of the SAD approach is to model an observation at time *t* by regression on the preceding observations. We chose to model a longitudinal trait with IGE using a SAD model because of the following advantages over the two main classical approaches used in genetic studies for longitudinal data, i.e. character process (CP) and random regression (RR) models: (1) the SAD approach can account for non-stationarity at the level of variances and correlations [14], in contrast to the CP approach, while the RR approach cannot model a situation with stationarity at the variance level and correlation between time points different than 1; and (2) extension of the SAD approach to the multiple-trait case is straightforward, in contrast to the CP approach, and requires fewer parameters than the RR approach [15]. The same applies to the case of correlated random effects in the analysis of just one phenotype, which is the case of correlated direct and indirect effects in our study.

## Methods

### Animals and housing conditions

Animals were raised on the experimental farm of IRTA in Spain between July 2012 and June 2014. A detailed description of the experiment is in Piles et al. [7]. In short, after weaning (32 d of age), kits were housed in cages (0.38 m^2^) of eight individuals and fed under a restricted feeding regime corresponding to 75% of ad libitum feed intake during 5 weeks. To obtain a feed restriction of 25%, the amount of food given during week *j* was computed as 0.75 times the average feed intake of contemporary kits that were on a full feeding regime during week *j *− 1, plus 10% to account for the estimated increase in feed intake of growing animals. For week 1, the amount of feed for the restricted feeding regime was computed from data recorded in previous experiments on the same line from animals on a full feeding regime raised during the same season (multiplied by 0.75, 10% increase not included). Feed (commercial pellets for rabbits with (weeks 1 to 4) or without (week 5) antibiotics, as detailed in Piles et al. [7]) was distributed once a day in a 3-place feeder. Water was available ad libitum (one nipple drinker per cage). Individual body weights (BW) were recorded at weaning and weekly after weaning. Average daily gain (g/d) for each week was calculated as the change in BW from the beginning to the end of the week divided by the number of days elapsed (7 ± 1 d). On the weighing day, information regarding the animal health status was also recorded. Groups with animals showing disease symptoms (not caused by antagonist behaviors) or groups who suffered death events during the week were discarded from the analyses. The final dataset comprised 11,255 ADG records from 3096 individuals born in 1106 litters. The pedigree included information on 7701 rabbits. Descriptive statistics for the weekly ADG records are in Table 1.

**Table 1:** Descriptive statistics for average daily gain (g/d)

Week	N	Mean	Standard deviation	Range
1	2687	26.51	10.25	[0.83, 70.71]
2	2624	37.54	11.49	[3.57, 76.25]
3	2280	42.02	11.10	[3.57, 82.14]
4	1944	40.11	13.27	[0.71, 89.00]
5	1720	41.20	10.90	[3.33, 87.14]

### Data analysis

Let *y*_*ilm*_(*w*_*j*_), be the ADG of animal *i* (1 ≤ *i* ≤ 3096), born in litter *l* (1 ≤ *l* ≤ 1106), raised in group *m* (1 ≤ *m* ≤ 387), during week (1 ≤ *j* ≤ 5). The linear mixed model used to study ADG was:

1 $$\begin{aligned} y_{ilm} \left( {w_{j} } \right) & = \mu_{i} \left( {w_{j} } \right) + DGE_{i} \left( {w_{j} } \right) + \mathop \sum \limits_{{k \in K_{i} }} IGE_{k} \left( {w_{j} } \right) \\ & \quad + l_{l} \left( {w_{j} } \right) + g_{m} \left( {w_{j} } \right) + p_{i} \left( {w_{j} } \right), \\ \end{aligned}$$

where *μ*_*i*_ (*w*_*j*_) represents the fixed effects at week *j*, *K*_*i*_ the set of the seven group mates of focal individual *i*, and *DGE*_*i*_ (*w*_*j*_), *IGE*_*k*_ (*w*_*j*_), *l*_*l*_ (*w*_*j*_), *g*_*m*_ (*w*_*j*_) and *p*_*i*_ (*w*_*j*_) are the direct genetic, indirect genetic, litter, group, and pseudo-permanent animal effects for week *w*_*j*_, respectively. The litter, group and pseudo-permanent random effects were independent from each other and distributed as: ${\mathbf{l}}\;\sim\;N\left( {0,{\mathbf{I}}_{\varvec{l}} \; \otimes \;\varvec{\Sigma}_{\varvec{l}} } \right),\;{\mathbf{g}}\;\sim\;N\left( {0,{\mathbf{I}}_{\varvec{g}} \; \otimes \;\varvec{\Sigma}_{\varvec{g}} } \right)$, and ${\mathbf{p}}\;\sim\;N\left( {0,{\mathbf{I}}_{\varvec{p}} \; \otimes \;\varvec{\Sigma}_{\varvec{p}} } \right)$, where ${\varvec{\Sigma}}_{\varvec{l}} ,\; {\varvec{\Sigma}}_{\varvec{g}}$ and ${\varvec{\Sigma}}_{\varvec{p}}$ are 5 × 5 covariance matrices corresponding to litter, group, and pseudo-permanent animal effects, respectively, for the 5-week period of observation, and ${\mathbf{I}}_{\varvec{l}} ,\;{\mathbf{I}}_{\varvec{g}}$ and ${\mathbf{I}}_{\varvec{p}}$ are identity matrices of appropriate size. Conversely, the direct and indirect genetic effects were correlated $$\left[ {\begin{array}{*{20}c} {{\mathbf{DGE}}} \\ {{\mathbf{IGE}}} \\ \end{array} } \right]\;\sim\;N\left( {0,{\mathbf{A}}\; \otimes \;{\varvec{\Sigma}}_{{\varvec{GE}}} } \right)$$, where ${\varvec{\Sigma}}_{{\varvec{GE}}}$ is the 10 × 10 (co)variance matrix for the genetic effects (5 weeks for the direct genetic effects and 5 weeks for the indirect genetic effects) and ${\mathbf{A}}$ is the additive relationship matrix based on pedigree. Possible non-null covariances between random effects at different times were taken into account using a SAD approach [16–18]. It should be noted that this model does not include a residual term to help convergence and to avoid identifiability problems between structured permanent and classical residual covariance matrices [19], as in previous studies using the SAD approach [15, 17]. Thus, the residual variance was by definition included in the (co)variance matrix of the pseudo-permanent animal effects of the model.

For a given random effect, independent from the other random effects of the model, ${\mathbf{p}}$ for instance, the general form of the SAD model of order *α* for animal *i* was: $p_{i} \left( {w_{j} } \right) = \sum\nolimits_{s = 1}^{\alpha } {\theta_{s,j} } p_{i} \left( {w_{j - s} } \right) + e_{p,i} \left( {w_{j} } \right)$, where *θ*_*sj*_ is the *s*th antedependence parameter for week *j*, and *e*_*p*,*i*_ (*w*_*j*_) is the error term, normally distributed with mean 0 and innovation variance *σ*_*p*_^2^ (*w*_*j*_). Parameters *θ*_*sj*_ and *σ*_*p*_^2^ (*w*_*j*_) were assumed to be continuous functions of time: $\theta_{s,j} = \sum\nolimits_{q = 0}^{{\beta_{s} }} {a_{sq} } w_{j}^{q}$, for a function of degree *β*_*s*_, and $\sigma_{p}^{2} \left( {w_{j} } \right)\, = \,exp\left( {\sum_{q = 0}^{\gamma } b_{q} w_{j}^{q} } \right)$, for a function of degree *γ*. The SAD models were then defined by the order of the antedependence (*α*), the degree of the polynomial for each antedependence parameter (*β*_1_ to *β*_*α*_), and the degree of the polynomial for the innovation variance (*γ*). We denote the different SAD models with those parameters as follows: ${\text{SAD}}\upalpha -\upbeta_{1} \ldots\upbeta_{\upalpha}\upgamma$. For instance, a SAD1-12 model corresponds to a SAD model with antedependence of order 1, and degrees 1 and 2 for the polynomial functions of the antedependence parameter and the innovation variance, respectively. A detailed description of this model is in Additional file 1.

In the case of correlated random effects (direct and indirect genetic effects), the dependence between the two terms was also considered using a SAD model, which is a particular case of the multiple-trait SAD model that was proposed by David et al. [20]. The general form of the SAD model for correlated effects of order *α* and *α*′ can be written as (for two effects, DGE and IGE, and for $j > { \hbox{max} }\left( {\alpha , \alpha^{\prime}} \right))$:

$$DGE_{i} \left( {w_{j} } \right) = \sum _{s = 1}^{\alpha } \theta_{sj} DGE_{i} \left( {w_{j - s} } \right) + \delta_{j} IGE_{i} \left( {w_{j} } \right) + e_{DGE,i} \left( {w_{j} } \right),$$

$$IGE_{i} \left( {w_{j} } \right) = \sum _{s = 1}^{{\alpha^{\prime } }} \theta_{sj}^{\prime } IGE_{i} \left( {w_{j - s} } \right) + e_{IGE,i} \left( {w_{j} } \right),$$

where notations are the same as for the SAD model for independent effects and *δ*_*j*_ is the cross-antedependence parameter at week *j*. The parameter *δ*_*j*_ was assumed to be a polynomial function of time.

The fixed effects included in the model and described in Piles et al. [7] were first selected step by step by comparing nested models using the likelihood ratio test. The fixed effects were week * body size (10 levels) and week * batch (70 levels) combinations, litter size (7 levels), and parity level (4 levels). After selection of fixed effects, the order and degree of the antedependence parameters were selected for each random effect in a model that assumed independence between direct and indirect genetic effects. Selection was performed by comparing nested models using the likelihood ratio test. Then, the correlation between direct and indirect genetic effects was included in the model. The degree of the cross-antedependence function (*δ*_*j*_) was also selected using the likelihood ratio test. The SAD model was fitted to the data using ASReml [21] and the OWN Fortran program that combines the single and multiple-SAD programs that we have recently developed (https://zenodo.org/record/192036 [20]), such that the two approaches can be applied in the same model, making it possible to consider cross-antedependence between direct and indirect genetic effects. The OWN program is freely available online at https://zenodo.org/record/1228058.

Once the SAD model has converged, parameter estimates (${\hat{\varvec{\upomega}}}$) can be used to compute estimates of the (co)variance matrix of the different random effects. For a given random effect, ${\hat{\varvec{\Sigma}}} = \left( {{\hat{\mathbf{L}}}^{\prime } \hat{\mathbf{D}}^{ - 1} {\hat{\mathbf{L}}}} \right)^{ - 1}$, where ${\hat{\mathbf{D}}}$ is a diagonal matrix with innovation variance estimates as components, ${\hat{\mathbf{L}}}$ is a lower triangular matrix with 1’s on the diagonal and negatives of the cross-antedependence parameter estimates below the diagonal entries [22].

The total phenotypic variance was calculated for each week *j* and for a group size of 8 as [23]:

$$\upsigma_{\text{Tj}}^{2} = \upsigma_{\text{DGEj}}^{2} + 7\upsigma_{\text{IGEj}}^{2} + 7r\left[ {2\upsigma_{\text{DGEj,IGEj}} + 6\upsigma_{\text{IGEj}}^{2} } \right] +\upsigma_{\text{lj}}^{2} +\upsigma_{\text{gj}}^{2} +\upsigma_{\text{pj}}^{2} ,$$

where $\upsigma_{\text{DGEj}}^{2}$, $\upsigma_{\text{IGEj}}^{2}$ and $\upsigma_{\text{DGEj,IGEj}}$ are the direct and indirect genetic variances and their covariance for week *j*, respectively, $\upsigma_{\text{lj}}^{2}$, $\upsigma_{\text{gj}}^{2}$ and $\upsigma_{\text{pj}}^{2}$ are the litter, group and pseudo-permanent variances for week *j*, respectively, and *r* is the average genetic relationship among cage mates, which was 0.16 in this study [7]. Estimates of direct, indirect and total heritabilities were computed for each week *j* as the ratio of the direct genetic, total indirect genetic ($49\upsigma_{\text{IGEj}}^{2}$ [1]) and total heritable variance ($\upsigma_{{{\text{TBV}}j}}^{2} =\upsigma_{\text{DGEj}}^{2} + 14\upsigma_{{{\text{DGE}}_{j} ,{\text{IGE}}_{j} }} + 49\upsigma_{{{\text{IGE}}j}}^{2}$ [24]), respectively, relative to the total phenotypic variance. To obtain standard errors of the co-variance and heritability estimates, we performed a multivariate normal sampling approach of ${\hat{\varvec{\upomega}}}$, as described in Houle and Meyer [25]: ${\tilde{\varvec{\omega} }}\;\sim\;N\left( {{{\hat{\varvec{\upomega} }}},\;\mathbf{H}\left( {{\hat{\varvec{\upomega }}}} \right)} \right)$, where $\mathbf{H}\left( {{\hat{\varvec{\upomega }}}} \right)$ is the inverse of the information matrix at convergence. We sampled $\tilde{\varvec{\omega }}$ 10,000 times, computed ${\tilde{\varvec{\Sigma }}}$ and heritabilities for each sample, and removed samples that led to a non-positive semi-definite matrix of ${\tilde{\varvec{\Sigma }}}$. Estimates of heritabilities and $\hat{\Sigma }$ were the mean of the heritabilities and ${\tilde{\varvec{\Sigma }}}$ across samples and their standard errors were the standard deviation of the samples. In addition, we calculated correlations between elements of ${\tilde{\varvec{\Sigma }}}$ to assess the estimability of the covariance components in this complex model.

### Simulation to assess response to selection

To evaluate the advantage of the SAD model with IGE for genetic selection of longitudinal traits that are affected by IGE in comparison with a model that ignores IGE, we performed a simulation study with genetic selection. The simulated population was a closed nucleus of discrete generations and constant size. The base population consisted of 30 unrelated sires and 120 unrelated dams. Each founder sire was mated to four founder females to give birth to 960 offspring (8 offspring per mating and a 50/50 sex ratio). Then, among the offspring, one male per sire and 120 females were randomly selected to be breeders of the next generation. During the genetic selection process over seven generations, the best male per sire (i.e. 30 males) and the best 120 females were selected at each generation to be breeders of the next generation. The choice of the “best” animals was based on their estimated breeding values (EBV) that were calculated according to the genetic evaluation strategies described below. Each selected male was randomly mated to four of the selected females, avoiding individuals from the same sire family.

The population and phenotypes were generated to match the previously described experimental data design. Individuals in each generation were assigned to groups of eight animals, with each group originating from four full-sib families and each family contributing two progeny. Individual phenotypes (5 records per animal, corresponding to 5 weeks of fattening) were constructed according to a multiple trait model that considered ADG as a different trait for each week. Average daily gain of each animal was computed for each week as the sum of: a week effect, the animal’s direct genetic effect for the corresponding week, the indirect genetic effects of the seven other pen mates for the corresponding week, a week-specific group effect, and a pseudo-permanent effect (correlated within animal): $y_{im} \left( {w_{j} } \right) = \mu_{i} \left( {w_{j} } \right) + DGE_{i} \left( {w_{j} } \right) + \sum\nolimits_{{k \in K_{i} }} {IGE_{k} } \left( {w_{j} } \right) + g_{m} \left( {w_{j} } \right) + p_{i} \left( {w_{j} } \right)$, (= Eq. (1) without litter effects). For each generation, multivariate normal distributions with unstructured covariance matrices were used to simulate the random effect values for each time point (genetic effects, group effect and pseudo-permanent effect). To stick as close as possible to reality, (co)variance values used in the unstructured matrices were close to those obtained with the SAD model in the data analysis step (detailed values in Additional file 2). For the genetic effects, to better assess the impact of the correlation between direct and indirect effects on selection response, we considered three sets of parameters for the genetic (co)variance matrix to mimic weak, moderate, and strong genetic antagonism between direct and indirect effects. For these sets, the genetic covariance matrix was the same as that obtained in the data analysis part of the study but three different sets of values were assigned to the direct–indirect covariances: values similar to those estimated in the data analysis part of the study (strong antagonism), those values divided by 2 (moderate antagonism) or by 4 (weak antagonism). Direct and indirect breeding values of the founders were simulated as for the other random effects. Then, the direct and indirect breeding values of an offspring were simulated as the average breeding value of its parents plus a Mendelian sampling deviation, which was sampled from a multivariate normal distribution with covariance corresponding to half of the genetic covariance matrix.

Three selection scenarios were investigated. In scenario (1), a SAD model that ignored IGE was used to predict genetic effects [$y_{im} \left( {w_{j} } \right) = \mu_{i} \left( {w_{j} } \right) + DGE_{i} \left( {w_{j} } \right) + g_{m} \left( {w_{j} } \right) + p_{i} \left( {w_{j} } \right)$, SAD1-11 for all random effect functions] and the sum of the weekly direct EBV was used as the selection criterion. In scenarios (2) and (3), a SAD model (same orders and degrees for the polynomial functions as those selected in the data analysis part of the study) with IGE was used to predict direct and indirect genetic effects for each time point [Eq. (1) with litter effects excluded]. The estimate of the total breeding value for each time point (i.e. weekly TEBV) was then computed as the sum of the direct EBV plus 7 times the indirect EBV of the corresponding time point leading to five TEBV per animal. In scenario (2), the selection criterion was the TEBV of the first week, while the sum of the weekly TEBV was used as the selection criterion in scenario (3). Variance components were estimated with the SAD model that included IGE [selection strategies (2) and (3)] or not [selection strategy (1)] using the 4800 records of the 960 offspring of the base population. Then, variance components were fixed to their estimates and considered as known to predict EBV in the different generations of the selection population.

The simulation was replicated 150 times. After all replicates were run, we assessed the capability of the SAD model with IGE to correctly account for the longitudinal structure of the data by assessing the standard deviation and the mean bias of each estimated variance component. Responses to the three selection strategies were compared based on the mean ADG in each generation.

## Results

### Parameter estimates

Mean ADG was 26.5 g/d for week 1, increased by 50% from week 1 to 3, and then remained constant (Table 1). This increase was associated with a small decrease in the coefficient of variation for ADG (38% for week 1 and  26% for week 5). Weekly ADG ranged from 0.7 to 89.0 g/d, which is quite large.

After selection of models, we retained the SAD1-11 model for all random effect functions and a polynomial function of degree 1 for the cross antedependence parameter *δ* that models the covariance between the direct and indirect genetic effects. The likelihood ratio test comparing this full SAD model to the same SAD model in which IGE were excluded was 40.52 (8 degrees of freedom).

**Fig. 1:**
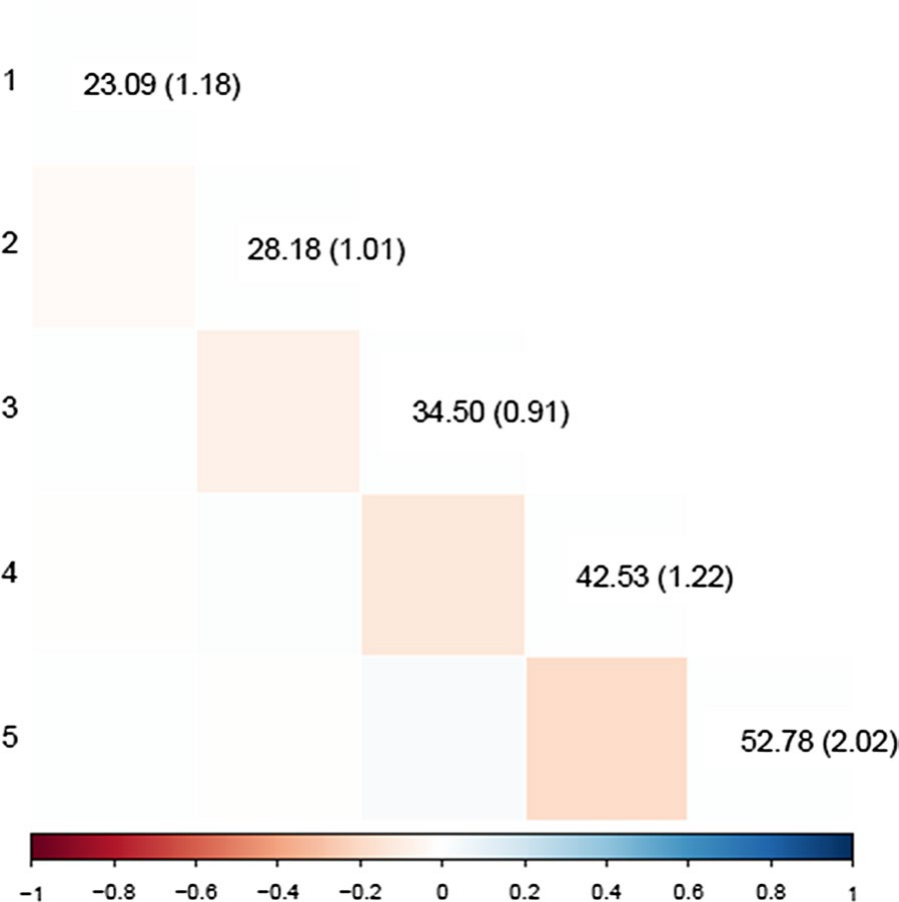
Estimates of pseudo-environmental variances for each week (on the diagonal, SE in brackets) and of correlations between weeks (below the diagonal.)

**Fig. 2:**
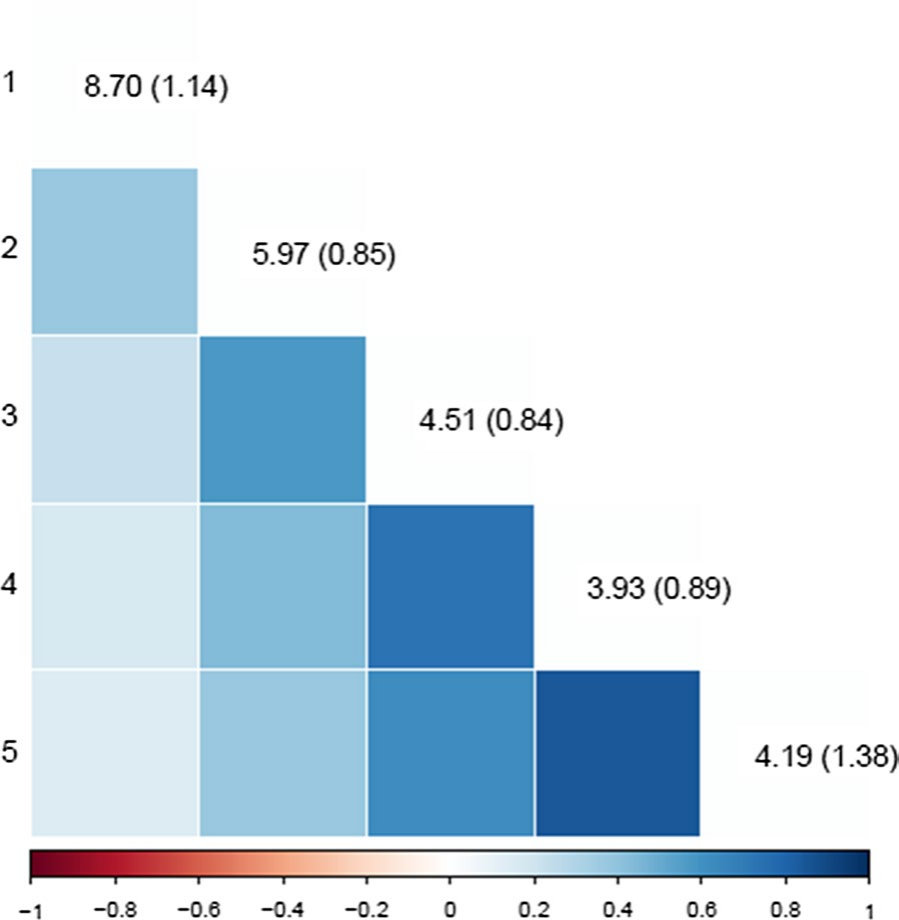
Estimates of litter variances for each week (on the diagonal, SE in brackets) and of correlations between weeks (below the diagonal)

**Fig. 3:**
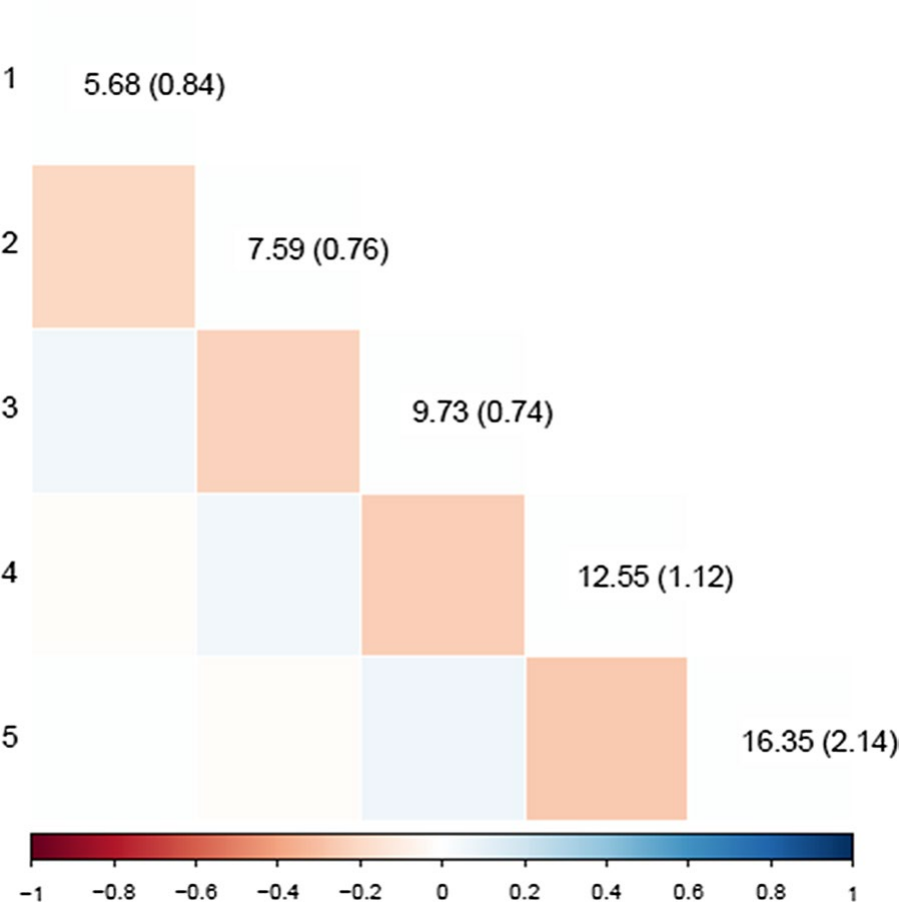
Estimates of group variances for each week (on the diagonal, SE in brackets) and of correlations between weeks (below the diagonal)

Estimates of variances of the pseudo-permanent, litter, and group effects for each week and their correlations between weeks that were obtained from the parameter estimates of the SAD model are presented in Figs. 1, 2 and 3, respectively. The pseudo-permanent effect variance increased with time from 23.1 to 52.8 (g/d)^2^. The ratio of the standard error to the pseudo-permanent effect variance was quite stable over time (~ 0.03). Estimates of correlations between the pseudo-permanent effects for different weeks were rather low and not significantly different from 0, except between consecutive weeks from week 2 to week 5, for which they were low and negative[− 0.08 (± 0.02) for weeks 2 to 3, − 0.13 (± 0.02) for weeks 3 to 4, and − 0.18 (± 0.03) for weeks 4 to 5]. The variance of litter effects decreased with time from 8.7 to 4.2 (g/d)^2^. Estimates of correlations between the litter effects for the different weeks were always positive and tended to decrease with the time interval between weeks. These correlations were higher between the four last weeks (ranging from 0.37 to 0.84) than between week 1 and the other weeks (ranging from 0.14 to 0.38). The estimate of the variance of the group effect increased with time from 5.7 to 16.4 (g/d)^2^. Estimates of correlations between the group effects of consecutive weeks tended to be negative (ranging from − 0.20 to − 0.26) and null otherwise.

**Fig. 4:**
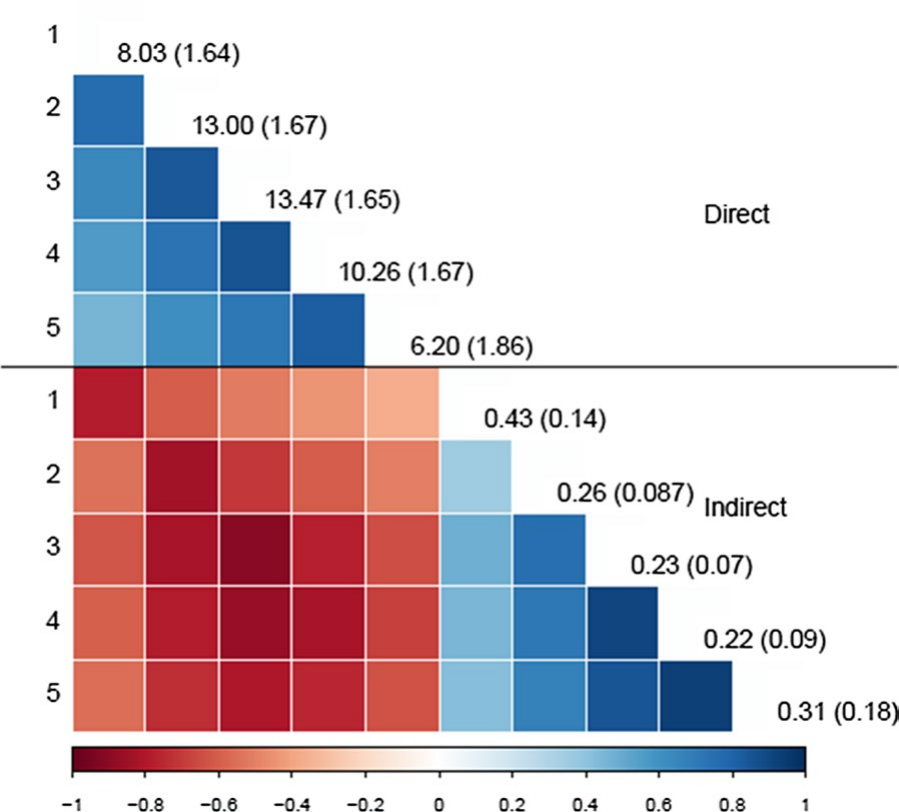
Estimates of genetic variances for each week (on the diagonal, SE in brackets) and of correlations between weeks and between direct and indirect genetic effects (below the diagonal)

Estimates of genetic variances and correlations are in Fig. 4. Estimates of genetic variances were not significantly different between consecutive weeks for both direct and indirect genetic effects. However, the observed general trend was an increase of the direct genetic variance from weeks 1 to 3 [from 8.03 to 13.47 (g/d)^2^] followed by a decrease [6.20 (g/d)^2^ at week 5], and a decrease of the indirect genetic variance [from 0.44 to 0.22 (g/d)^2^] from weeks 1 to 4. Then, for week 5, the indirect genetic variance increased but the associated standard error was substantial [0.31 (± 0.18)]. Estimates of correlations between the direct genetic effects for different weeks were moderate to high (ranging from 0.46 to 0.86) and tended to decrease with the time interval between measurements. The same trend was observed for the indirect genetic effects for weeks 2 to 5 (correlations ranging from 0.62 to 0.91). Estimates of correlations of indirect genetic effects of the first week with those of other weeks did not follow the same pattern and were weaker (ranging from 0.33 to 0.47). Estimates of genetic correlations between direct and indirect effects were negative within (ranging from − 0.57 to − 0.89) and between weeks (ranging from − 0.33 to − 0.84).

Heritability estimates are in Table 2. Estimates of direct heritability were moderate for weeks 1 to 4, ranging from 0.15 to 0.24, and its associated standard error was stable over time (0.02 to 0.03). For week 5, the estimate of direct heritability was lower [0.08 (± 0.02)]. The estimate of the heritability of indirect genetic effects ($49\upsigma_{\text{IGEj}}^{2} / {\text{total}}\;{\text{phenotypic}}\;{\text{variance}}\;{\text{in}}\;{\text{week}}\;{\text{j}})$ was moderate (ranging from 0.16 to 0.44). Although estimates of indirect heritability were not significantly different between weeks, we observed that this heritability was 2.5 times higher for week 1 than for the other weeks. Estimates of total heritability were low to moderate (ranging from 0.05 to 0.19) and were associated with large standard errors (0.04 to 0.10). Although estimates of total heritability were not significantly different between weeks, we observed that it tended to decrease from week 1 to 4.

**Table 2:** Heritability estimates for average daily gain across weeks

Week	Direct heritability	Indirect heritability	Total heritability
1	0.17 (0.03)	0.44 (0.13)	0.19 (0.10)
2	0.24 (0.03)	0.23 (0.07)	0.09 (0.06)
3	0.22 (0.02)	0.18 (0.06)	0.05 (0.04)
4	0.15 (0.02)	0.16 (0.06)	0.06 (0.04)
5	0.08 (0.02)	0.18 (0.11)	0.12 (0.10)

### Estimation efficiency of variance components

Estimates of variance components converged for both SAD models (with or without IGE) for only a percentage of the simulation replicates (for 79, 77 and 48% of the replicates for the scenarios with weak, moderate, and strong simulated antagonism between direct and indirect genetic effects, respectively). Consequently, replicates for which one of the two SAD models did not converge were removed from the analysis. Table 3 includes the bias and variability over the replicates that converged for each variance component from the SAD model that included IGE. Bias and variability of variance components were not affected by size of the genetic antagonism between direct and indirect effects, except for estimates of the indirect genetic variance and indirect genetic correlation. Coefficients of variation ($\frac{{std\left( {\hat{\omega }} \right)}}{{E\left( {\hat{\omega }} \right)}}*100\%$) were in the same range for the direct genetic and group variances (~ 19 and 17%, respectively) but were higher for the indirect genetic variances and increased from 39 to 58% as the genetic antagonism decreased. The direct genetic variance was correctly estimated (the mean relative bias, $\left( {E\left( {\hat{\omega }} \right) - \omega } \right)/\omega$, ranged from 1 to 2%), while the group variance tended to be slightly underestimated (mean relative bias ~ − 4%) and the indirect genetic variance was overestimated (mean relative bias ranging from 18 to 27%). All correlations were correctly estimated, with a bias close to 0, except the indirect genetic correlation, which was slightly overestimated (bias ranging from − 0.09 to − 0.03). The standard deviations of the group and the direct genetic correlations were in the same range (~ 0.07), while the standard deviation for the direct–indirect genetic correlation was higher (~ 0.15) and the standard deviation for the indirect genetic effects was the highest (~ 0.26).

**Table 3:** Average and range of bias and dispersion of the variance component estimates obtained with a SAD model with IGE applied to the simulated longitudinal data

Component	Criterion^a^	Simulated antagonism between direct and indirect genetic effects		
		Strong (48%)^b^	Moderate (77%)^b^	Weak (79%)^b^
Direct genetic variance	Relative bias	2 [0–4]	1 [0–2]	2 [1–3]
	CV	18 [13–25]	20 [14–30]	20 [14–32]
Direct genetic correlation	Bias	0.01 [0–0.03]	0.01 [0.00–0.02]	0.01 [0.00–0.02]
	SD	0.07 [0.04–0.11]	0.07 [0.04–0.11]	0.08 [0.04–0.11]
Indirect genetic variance	Relative bias	27 [19–43]	19 [11–28]	18 [5–33]
	CV	39 [35–48]	52 [47–61]	58 [48–63]
Indirect correlation	Bias	− 0.03 [− 0.07 to 0.02]	− 0.09 [− 0.12 to 0.05]	− 0.08 [− 0.12 to 0.07]
	SD	0.20 [0.15–0.30]	0.27 [0.22 − 0.35]	0.31 [0.27–0.39]
Direct–indirect correlation	Bias	0.04 [− 0.01 to 0.14]	0.00 [− 0.08 to 0.08]	0.00 [− 0.06 to 0.08]
	SD	0.13 [0.08–0.25]	0.17 [0.11–0.25]	0.18 [0.09–0.26]
Group variance	Relative bias	− 4 [− 6 to − 3]	− 5 [− 7 to − 2]	− 4 [− 7 to 0]
	CV	17 [13–24]	17 [13–25]	18 [13–28]
Group correlation	Bias	0.00 [− 0.03 to 0.03]	0.00 [− 0.03 to 0.03]	0.00 [− 0.02 to 0.02]
	SD	0.06 [0.01–0.12]	0.07 [0.01–0.16]	0.08 [0.01–0.17]

The average and the range is given for each criteria (average over five components for the variance (5 weeks), over 10 (or 25) components for correlations)

*CV* coefficient of variation, *SD* standard deviation

^a^For variance component *ω*, bias is evaluated by the mean relative bias $\frac{{E\left( {\hat{\omega }} \right) - \omega }}{\omega }*100)$ and dispersion by the coefficient of variation ($\frac{{std\left( {\hat{\omega }} \right)}}{{E\left( {\hat{\omega }} \right)}}*100)$. For correlations *ω*, bias is evaluated by the mean bias ($E\left( {\hat{\omega }} \right) - \omega$) and dispersion by the standard deviation

^b^Percentage of replicates used

### Response to selection

**Fig. 5:**
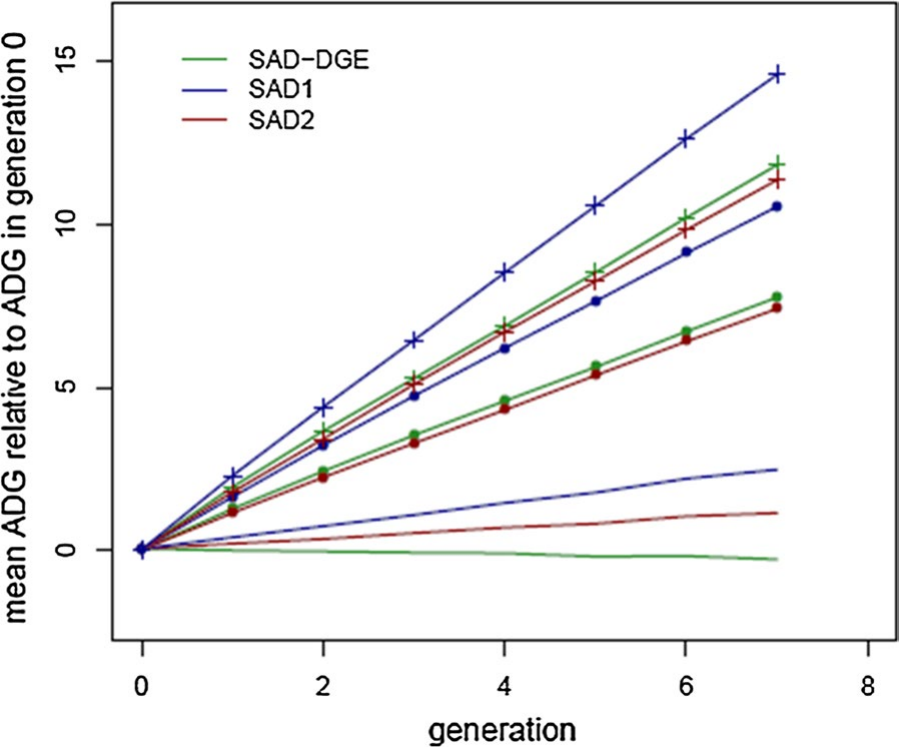
Changes in the mean ADG across generations for each selection strategy. SAD–DGE-selection criterion: sum of the weekly direct EBV obtained with a SAD model without IGE; SAD1-selection criterion: sum of the weekly TEBV obtained with a SAD model with IGE; SAD2-selection criterion: TEBV of the first week obtained with a SAD model with IGE. Straight line, line with (dot) and line with + represent strong, moderate and weak simulated genetic antagonism between direct and indirect effects, respectively

Changes in the mean ADG per generation are shown in Fig. 5 for the three selection strategies and the three sets of genetic antagonism between direct and indirect effects. For the three scenarios, the mean ADG increased linearly with generation when a SAD model including IGE was used to predict EBV. For the three sets of genetic antagonism between direct and indirect effects, the increase was highest when selection was performed using the sum of the weekly TEBV obtained with a SAD model that included IGE. Selection on the TEBV of week 1 obtained with a SAD model that includes IGE or on the direct EBV obtained with a SAD model that ignores IGE resulted in similar increases in ADG per generation, except when a strong genetic antagonism between direct and indirect effects was simulated. In that case, the model with IGE outperformed the model without IGE, for which there was no response to selection. For all selection strategies, response in ADG declined as the genetic antagonism between direct and indirect effects increased.

**Fig. 6:**
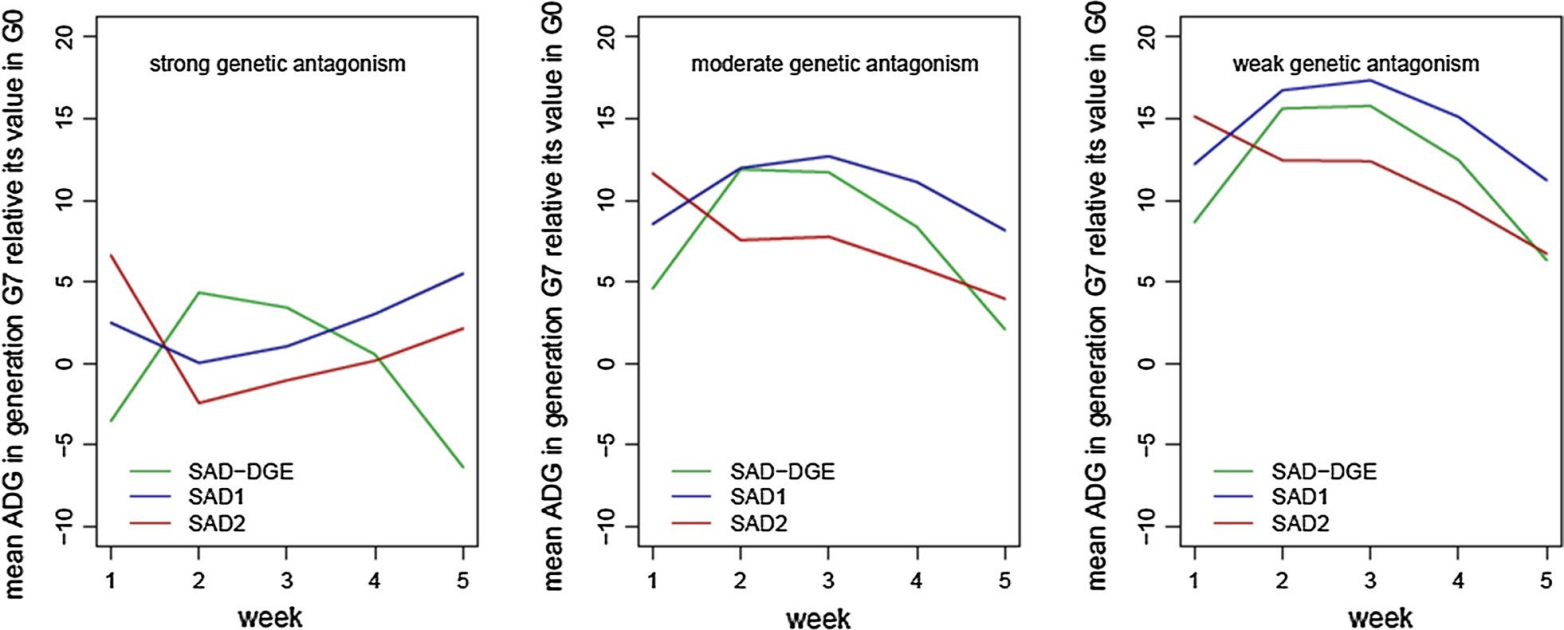
Mean ADG by week in the last generation for each selection strategy and for different sets of simulated genetic antagonism between direct and indirect genetic effects. SAD–DGE-selection criterion: sum of the weekly direct EBVs obtained with a SAD model without IGE; SAD1-selection criterion: sum of the weekly TEBV obtained with a SAD model with IGE; SAD2-selection criterion: TEBV of the first week obtained with a SAD model with IGE

The effects of the different selection strategies and scenarios across the different weeks of growth were also explored by assessing the mean ADG by week in the 7th generation, relative to the base generation (Fig. 6). When a weak to moderate genetic antagonism between direct and indirect effects was simulated, the same pattern was observed: ADG increased for all weeks and for all selection strategies. As expected, response to selection at week 1 was higher with selection on the TEBV of week 1 obtained with a SAD model that included IGE. The pattern was different with a strong genetic antagonism between direct and indirect effects. On the one hand, with selection on the sum of the weekly TEBV obtained with a SAD model with IGE, the mean ADG increased for weeks 1, 3, 4 and 5, while no changes were observed for week 2. On the other hand, with selection on the TEBV of week 1 obtained with a SAD model with IGE, there was a high increase in the mean ADG for week 1, a clear decrease for weeks 2 and 3, no changes for week 4, and a moderate increase for the last week of observation. When a SAD model without IGE was used to select animals, the pattern was the opposite (increases in weeks 2 and 3 and reductions in weeks 1 and 5).

## Discussion

One of the characteristics of the analysis of longitudinal data is the within-subject correlation of the measurements (in addition to between-subject correlations due to shared genetic or environmental factors). A large number of parameters (100) are necessary to model the covariance structure of our data using an unstructured model with the same random effects as described here. Several flexible approaches exist to model the covariance structure of the data with a reduced number of parameters, such as random regression (RR), SAD [26], and character process (CP) models [16, 27]. Among these, we chose the SAD approach for several reasons: (1) it relaxes the stationary correlations assumption made in CP models [28]; (2) it has been shown to better fit the data compared to the RR model in many situations [16–18, 20]; (3) it is less sensitive to the drawbacks reported for the RR model, such as border effects [29] and inability to properly estimate correlations that decrease rapidly over time [16]; and (4) it generally requires fewer parameters than the CP model to model covariance structures. The SAD approach has been used to perform genetic studies of several longitudinal traits, such as repeated measurements of weight, feed intake, reproduction traits [17, 18, 20]. It has also been used to model residual (co)variances in models for quantitative trait loci (QTL) detection in the framework of functional mapping [30] of longitudinal traits in animals [31] and plants [32]. To our knowledge, this is the first time that a SAD approach has been used in quantitative genetic mixed models to model a trait with indirect genetic effects. It should be noted that we tried to apply a RR model to these data using the REML approach but the estimation procedure never converged.

The mean weekly ADG reported here are consistent with those reported over the whole fattening period under a feed restriction of 80% by Drouilhet et al. [33] (40.4 g/d). Because of the limited amount of food, the ADG of restricted animals was lower than that of animals fed ad libitum from the same population of rabbits [7]. In addition, there was an effect of feeding restriction on the growth pattern, with the deceleration phase and the inflexion point of the growth curve being delayed. Because animals were raised in collective cages, we included indirect genetic effects in the model to account for the competition between individuals to access feed. In a previous analysis of the same dataset that did not consider the longitudinal characteristics of the data, Piles et al. [7] confirmed that indirect genetic effects play an important role in ADG under restricted feeding. The significance of IGE was also confirmed in our study when the longitudinal characteristics of the data were accounted for by the likelihood ratio test that compared the SAD model with and without IGE. The importance of indirect genetic effects on the ADG of group-housed animals has also been reported in other species such as pigs [34, 35] and in other breeding conditions (e.g. conventional barren *vs* enriched pens [36]).

**Fig. 7:**
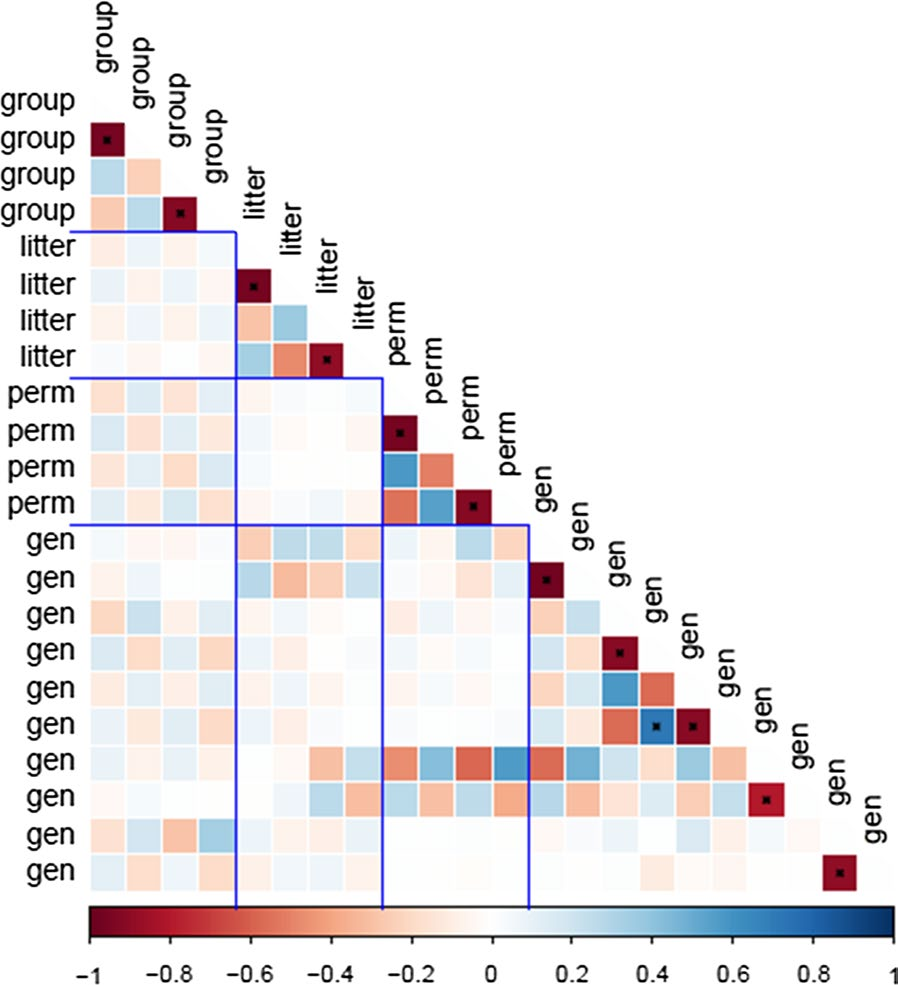
Correlation matrix between parameter estimates. For group, litter and permanent random effects (“perm”); parameters are presented in the following order: the two regression coefficients of the antedependence parameters, then the two regression coefficients of the innovation variance. For the genetic effects (“gen”), the parameters are presented in the following order: the first six parameters correspond to the regression coefficients of the antedependence parameters for the direct (first and second parameters), indirect (third and fourth parameters) effects, and the regression coefficients of the cross-antedependence parameters (fifth and sixth parameters). The four last parameters correspond to the regression coefficient for the innovation variance of the direct (seventh and eighth parameters) and indirect (ninth and tenth parameters) genetic effects. Cell with a (dot) indicates an absolute correlation value higher than 0.6

Estimating IGE can be challenging [37] because the statistical power for detecting IGE is determined by the group-population structure [38, 39] and there is a risk of confounding IGE with environmental (i.e. group) effects, leading to non-identifiability of the (co)variance components of the social model [40]. Even if the structure of the design in our study (small group size, large number of groups and 4 * 2 full-sibs per group) meets the requirements to detect IGE and a group effect was included in the model as a random effect to help identifiability of parameters, identifiability of the (co)variance components is not guaranteed [40]. Exploring the information matrix ${\mathbf{I}}\left( {{\hat{\varvec{\upomega}}}} \right)$ helps to detect identifiability problems for an unknown parameter vector ${\varvec{\upomega}}$ [41]. The condition number (square root of the ratio of the first to the last eigenvalue) of the information matrix ${\mathbf{I}}\left( {{\hat{\varvec{\upomega}}}} \right)$ of the SAD model used here was large (512), which may lead to the occurrence of identifiability problems. Nonetheless, the condition number of the submatrix of ${\mathbf{I}}\left( {{\hat{\varvec{\upomega}}}} \right)$ that considered only the first regression coefficients of the SAD functions for all random terms (11 * 11 matrix) was equal to 35, which indicates that dependencies of parameter estimates were not between parameters of different random effects. In fact, close inspection of the correlation matrix among parameter estimates (Fig. 7, ${\mathbf{H}}\left( {{\hat{\varvec{\upomega}}}} \right) = {\mathbf{I}}\left( {{\hat{\varvec{\upomega}}}} \right)^{ - 1}$) showed that correlation between regression coefficients within polynomial functions were high, while correlations between SAD parameters related to different random effects were low. For instance, the correlations between parameter estimates for the genetic effects and those related to the group effect were moderately high (ranging from −  0.57 to 0.56, and averaging 0.14), which indicates that the information provided by the data was sufficient to disentangle common environment effects from indirect genetic effects in the SAD model. To better understand the relationship between variance components of the random effects obtained with estimates of the SAD model, we also computed the correlation between estimates at the level of the (co)variance components using the 10,000 random samples of $\tilde{{\varvec{\upomega}}}$ in $\tilde{{\varvec{\upomega}}}\sim{\text{N}}\left( {{\hat{\varvec{\upomega}}},{\text{H}}\left( {{\hat{\varvec{\upomega}}}} \right)} \right)$. The corresponding 100 * 100 matrix is shown in Additional file 3. Correlations between (co)variance component estimates of different random effects were generally low. Those of the five indirect genetic variances with the five group variance estimates were less than 0.27 (in absolute value), while those of the 25 direct–indirect genetic covariances with the group (co)variance estimates were less than 0.18 (in absolute value). Such results favor the possibility to separate the variance estimates of the different random effects in the model.

The relative importance of the litter effect was moderate, in accordance with previous studies [42, 43]. It decreased with time (from 18 to 5% of the total variance), probably as a consequence of the well-known decrease over time of the maternal influence on progeny performance [44, 45]. The relative importance of the group effect (approximately 15% of the total variance) was in line with previous results in other species (18% [35]). Because residuals were assumed to be independent among the individuals of a given group, the variance of the group effect included in the model originates from both social interactions between individuals (correlated residual effects) and physical differences between pens (location in the building for instance) [46]. Accounting for the environmental dependence between group mates in this manner is possible if, and only if, the covariance among residuals of group mates is positive [23]. This condition was verified week by week for this population in a previous study (correlation between residuals of group mates over weeks were 0.11, 0.17, 0.19, 0.18 for weeks 1 to 4, respectively) [7]. The slight increase in the relative importance of the group effect over the study period (from 11 to 20% of the total variance) could be related with this previously reported slight increase in the weekly correlation between residuals of group mates.

We obtained moderate estimates of the direct heritability, which is consistent with previous estimates reported in the literature for the same species 0.22 [33] and 0.18 [47]. The decrease in the direct genetic variance, and thus in heritability, at week 5 is probably related to the change in feed composition that occurred that particular week.

The moderate heritability of indirect genetic effects is in line with those reported in previous studies for pigs (~ 0.13 [48] and ~ 0.39 [23]). It is interesting to note that the indirect genetic effects were higher for week 1, which is in accordance with studies on the behavior of animals raised in groups that report a higher level of aggressiveness at mixing, which reflects the establishment of social hierarchy that generally occurs within 3 days after mixing. In the same population as used here, Dalmau et al. [13] observed signs of antagonistic behavior such as biting, displacement, and animals jumping on top of each other over the whole growing period but especially during week 1. In mice and pigs, indirect genetic effects are known to induce antagonistic behavior at mixing [49, 50]. Furthermore, we observed that the genetic correlation of the IGE in week 1 with the IGE in other weeks was lower than the correlation between IGE in weeks 2, 3, 4, and 5, which also indicates that IGE expressed just after mixing is a different trait from IGE expressed later in life.

The genetic correlation between direct and indirect genetic effects was negative regardless of the week considered, which indicates that individuals with a positive (direct) breeding value for their own growth tend to have a negative (indirect) breeding value for the growth of their cage mates and vice versa. This is consistent with the idea of competition between animals for a fixed amount of feed. Nonetheless, although we found no evidence of lack of identifiability for the parameters in our analysis, we suspect that the estimates of the genetic correlations between direct and indirect genetic effects are higher than their real value. An antagonism probably exists between direct and indirect genetic effects but not with such a strong negative correlation. The antagonism of these two effects has also been reported in previous studies on restricted feeding. Muir [51] described a moderate to high negative (− 0.56) genetic correlation between direct and indirect genetic effects on weight at 6 weeks of age of Japanese quail in groups of 16 birds. However, Bergsma et al. [23] found a neutral relationship between direct and indirect genetic effects for pigs in groups of 6 to 12 pen mates of the same sex.

Based on our results, we hypothesize that the growth of animals under a restricted feeding regime can be improved and that the delay of their growth curve can be shortened by reducing the negative impact of indirect effects, especially during the first week after mixing. One way to do this is to provide an enabling environment that decreases indirect effects by limiting the feed restriction during the first week after mixing (85 instead of 75% of ad libitum feed intake instead, for instance). Currently, this strategy is applied on several rabbits farms (Tudela F., personal communication). Another strategy is to perform genetic selection to improve the ADG, as investigated here by simulation.

For the simulations, unstructured (co)variance matrices were used to simulate the data and the SAD model was used to estimate variance components for different weeks. To obtain convergence of complex SAD models, such as those used in this study; the most appropriate method involves starting with a simple SAD model (low antedependence orders and low degrees for the polynomial functions) and to increase the degree and order step-by-step by using estimates of the preceding reduced model as starting values. For the simulations, we did not use this method (because it required too much computing time) and applied the full SAD models right away. This explains the lack of convergence of the SAD model for several replicates. However, it should be noted that the percentage of replicates that did not converge increased with the degree of genetic antagonism between direct and indirect genetic effects. This confirms that when one parameter (the genetic correlation in our case) is close to the boundary of the parameter space, estimation of model parameters becomes difficult. Using a larger dataset is one approach to overcome this problem [52].

Across the replicates that converged, the bias in estimates of the variance components and the correlations between weeks were moderate, which indicates that the SAD model was able to estimate the different variance components. Nonetheless, it should be noted that the indirect genetic variances tended to be overestimated, while the group variances were slightly underestimated, which confirms the difficulties (but not the impossibility) of separating IGE from group effects. Thus, in practice, when applying such a complex model, it is highly recommended to inspect closely the average information matrix to determine whether the data structure supports the fitted model.

We proposed three selection scenarios. All scenarios used the SAD model to compute TEBV and took the longitudinality of the data and the possibility that ADG corresponds to different traits at different time points into account. In scenario (1), IGE were not accounted for in the SAD model. As expected, applying a model with IGE for selection led to a larger increase in ADG than when IGE were ignored in the evaluation model [comparison of selection strategies (1) and (3)]. The benefit of including IGE in the evaluation model by simulation was reported previously [53]. The difference between the two selection strategies decreased as the genetic antagonism between direct and indirect effects decreased and is expected to be low in case of cooperation instead of competition between animals. In the extreme case of a strong direct–indirect genetic antagonism, the mean ADG was not improved by selection if IGE were not considered in the evaluation model.

Based on the five TEBV per animal (5 weeks), different selection indexes can be computed, with different weights for each week [selection strategies (2) and (3)]. In scenario (2), since the most important negative antagonistic behaviors between cage mates occurred during the first week after mixing, the weight was 1 for week 1 and 0 for the other weeks. In scenario (3), we considered equal weights for all weeks. Response to selection for scenario (2) was as expected: a strong increase in ADG for the first week after mixing. However, this response was associated with a detrimental effect on ADG for the two following weeks when data were simulated with a strong genetic antagonism between direct and indirect genetic effects. Such negative responses for weeks 2 and 3 were the consequence of negative correlations of the TEBV for week 1 with those of weeks 2 or 3 (− 0.30 and − 0.07, respectively), while the correlation was null with week 4 and slightly positive with week 5 (0.05). To avoid this detrimental correlated response, the selection criterion should take all weeks or at least weeks 1 and 2 into account. When the genetic antagonism between direct and indirect genetic effects was weak to moderate, correlations between TEBV for different weeks were positive and the detrimental effects for ADG in weeks 2 and 3 were not observed. However, using equal weights for all weeks in the selection criterion resulted in a higher mean ADG over weeks than when the TEBV of only the first week was used for selection regardless of the importance of the genetic antagonism between direct and indirect genetic effects. The selection scenarios (2) and (3) reflected two extreme strategies but it would be possible to refine the selection index by considering other sets of weights for the different weeks. It would probably be beneficial to increase the weight for week 2 to avoid a decrease in ADG by applying selection for this particular week in case of a strong genetic antagonism between direct and indirect genetic effects, or to build a selection index as a combination of the TEBV for week 1 and the direct EBV for the other weeks.

Note that the simulations were performed under the assumption that there were no genotype-by-environment interaction within a week, i.e. the direct and indirect genetic effects of an animal were the same regardless of the direct and indirect genetic effects of its group mates. This assumption may be questionable.

## Conclusions

Using a SAD model, we showed that IGE that act on ADG vary over time and that IGE are more important during the first week after mixing. Combining TEBV obtained from a SAD model that includes IGE in a selection index is the most effective strategy to improve longitudinal ADG when IGE occur.

## Additional files

**Additional file 1.** Detailed description of a SAD1-12 model.

**Additional file 2.** Variance components used for the simulation.

**Additional file 3.** Correlation matrix among (co)variance component estimates.

## References

[1] Bouwman AC, Bergsma R, Duijvesteijn N, Bijma P (2010). Maternal and social genetic effects on average daily gain of piglets from birth until weaning. J Anim Sci.

[2] Ellen ED, Visscher J, van Arendonk JA, Bijma P (2008). Survival of laying hens: genetic parameters for direct and associative effects in three purebred layer lines. Poult Sci.

[3] Camerlink I, Turner SP, Bijma P, Bolhuis JE (2013). Indirect genetic effects and housing conditions in relation to aggressive behaviour in pigs. PLoS One.

[4] Alemu SW, Bijma P, Møller SH, Janss L, Berg P (2014). Indirect genetic effects contribute substantially to heritable variation in aggression-related traits in group-housed mink (*Neovison vison*). Genet Sel Evol.

[5] Griffing B (1967). Selection in reference to biological groups I. Individual and group selection applied to populations of unordered groups. Aust. J Biol Sci.

[6] Wolf JB, Brodie ED, Cheverud JM, Moore AJ, Wade MJ (1998). Evolutionary consequences of indirect genetic effects. Trends Ecol Evol.

[7] Piles M, David I, Ramon J, Canario L, Rafel O, Pascual M (2017). Interaction of direct and social genetic effects with feeding regime in growing rabbits. Genet Sel Evol.

[8] Bijma P, Wade M (2008). The joint effects of kin, multilevel selection and indirect genetic effects on response to genetic selection. J Evol Biol.

[9] Estevez I, Andersen IL, Nævdal E (2007). Group size, density and social dynamics in farm animals. Appl Anim Behav Sci.

[10] Rodenburg TB, Koene P (2007). The impact of group size on damaging behaviours, aggression, fear and stress in farm animals. Appl Anim Behav Sci.

[11] Fraser D (1984). The role of behavior in swine production: a review of research. Appl Anim Ethol.

[12] Petersen HV, Vestergaard K, Jensen P (1989). Integration of piglets into social groups of free-ranging domestic pigs. Appl Anim Behav Sci.

[13] Dalmau A, Abdel-Khalek A, Ramon J, Piles M, Sanchez J, Velarde A (2015). Comparison of behaviour, performance and mortality in restricted and ad libitum-fed growing rabbits. Animal.

[14] Nunez-Anton V, Zimmerman DL (2000). Modeling non-stationary longitudinal data. Biometrics.

[15] Jaffrézic F, Thompson R, Hill WG (2003). Structured antedependence models for genetic analysis of repeated measures on multiple quantitative traits. Genet Res.

[16] Jaffrézic F, Pletcher SD (2000). Statistical models for estimating the genetic basis of repeated measures and other function-valued traits. Genetics.

[17] Jaffrézic F, Venot E, Laloe D, Vinet A, Renand G (2004). Use of structured antedependence models for the genetic analysis of growth curves. J Anim Sci.

[18] David I, Ruesche J, Drouilhet L, Garreau H, Gilbert H (2015). Genetic modeling of feed intake. J Anim Sci.

[19] Wang W (2013). Identifiability of linear mixed effects models. Electron J Stat.

[20] David I, Garreau H, Balmisse E, Billon Y, Canario L (2017). Multiple-trait structured antedependence model to study the relationship between litter size and birth weight in pigs and rabbits. Genet Sel Evol.

[21] Gilmour AR, Gogel BJ, Cullis BR, Thompson R (2009). ASReml user guide release 3.01.

[22] Pourahmadi M (1999). Joint mean-covariance models with applications to longitudinal data: unconstrained parameterisation. Biometrika.

[23] Bergsma R, Kanis E, Knol EF, Bijma P (2008). The contribution of social effects to heritable variation in finishing traits of domestic pigs (*Sus scrofa*). Genetics.

[24] Bijma P (2011). A general definition of the heritable variation that determines the potential of a population to respond to selection. Genetics.

[25] Houle D, Meyer K (2015). Estimating sampling error of evolutionary statistics based on genetic covariance matrices using maximum likelihood. J Evol Biol.

[26] Zimmerman DL, Nunez-Anton VA (2010). Antedependence models for longitudinal data.

[27] Speidel SE, Enns RM, Crews DH (2010). Genetic analysis of longitudinal data in beef cattle: a review. Genet Mol Res.

[28] Pletcher SD, Geyer CJ (1999). The genetic analysis of age-dependent traits: modeling the character process. Genetics.

[29] Druet T, Jaffrézic F, Boichard D, Ducrocq V (2003). Modeling lactation curves and estimation of genetic parameters for first lactation test-day records of French Holstein cows. J Dairy Sci.

[30] Wu R, Lin M (2006). Functional mapping-how to map and study the genetic architecture of dynamic complex traits. Nat Rev Genet.

[31] Zhao W, Chen YQ, Casella G, Cheverud JM, Wu R (2005). A non-stationary model for functional mapping of complex traits. Bioinformatics.

[32] Zhao W, Hou W, Littell RC, Wu R (2005). Structured antedependence models for functional mapping of multiple longitudinal traits. Stat Appl Genet Mol Biol.

[33] Drouilhet L, Gilbert H, Balmisse E, Ruesche J, Tircazes A, Larzul C, Garreau H (2013). Genetic parameters for two selection criteria for feed efficiency in rabbits. J Anim Sci.

[34] Canario A, Bijma P. Pig growth is affected by social genetic effects and social litter effects that depend on group size. In: Proceedings of the 9th world congress on genetic applied to livestock production, pp. 1–6 August 2010; Leipzig; 2010.

[35] Chen CY, Kachman SD, Johnson RK, Newman S, Van Vleck LD (2008). Estimation of genetic parameters for average daily gain using models with competition effects. J Anim Sci.

[36] Camerlink I, Bolhuis J, Duijvesteijn N, Van Arendonk J, Bijma P (2014). Growth performance and carcass traits in pigs selected for indirect genetic effects on growth rate in two environments. J Anim Sci.

[37] Bijma P (2014). The quantitative genetics of indirect genetic effects: a selective review of modelling issues. Heredity (Edinb).

[38] Bijma P (2010). Estimating indirect genetic effects: precision of estimates and optimum designs. Genetics.

[39] Cheng J, Buys N, Janssens S (2009). Full sib pens of pigs are not suitable to identify variance component of associative effect: a simulation study using Gibbs sampling. BMC Genet.

[40] Cantet RJC, Cappa EP (2008). On identifiability of (co)variance components in animal models with competition effects. J Anim Breed Genet.

[41] Jiang J (1996). REML estimation: asymptotic behavior and related topics. Ann Stat.

[42] Johnson ZB, Chewning JJ, Nugent RA (2002). Maternal effects on traits measured during postweaning performance test of swine from four breeds. J Anim Sci.

[43] Nagy I, Farkas J, Bíró-Németh E, Radnai I, Szendro Z (2011). Stability of estimated breeding values for average daily gain in Pannon White rabbits. Czech J Anim Sci.

[44] Cundiff LV (1972). The role of maternal effects in animal breeding: VIII. Comparative aspects of maternal effects. J Anim Sci.

[45] Zhang S, Bidanel JP, Burlot T, Legault C, Naveau J (2000). Genetic parameters and genetic trends in the Chinese × European Tiameslan composite pig line. I. Genetic parameters. Genet Sel Evol.

[46] Bijma P, Muir VM, Ellen ED, Wolf JB, van Arendonk JAM (2007). Multilevel selection 2: estimating the genetic parameters determining inheritance and response to selection. Genetics.

[47] Lavara R, Vicente J, Baselga M (2011). Genetic parameter estimates for semen production traits and growth rate of a paternal rabbit line. J Anim Breed Genet.

[48] Canario L, Lundeheim N, Bijma P (2017). The early-life environment of a pig shapes the phenotypes of its social partners in adulthood. Heredity (Edinb)..

[49] Canario L, Turner SP, Roehe R, Lundeheim N, D’Eath RB, Lawrence AB (2012). Genetic associations between behavioral traits and direct-social effects of growth rate in pigs. J Anim Sci.

[50] Wilson AJ, Gelin U, Perron MC, Réale D (2009). Indirect genetic effects and the evolution of aggression in a vertebrate system. Proc Biol Sci.

[51] Muir WM (2005). Incorporation of competitive effects in forest tree or animal breeding programs. Genetics.

[52] Bentler PM, Chou CP (1987). Practical issues in structural modeling. Sociol Methods Res.

[53] Liu J, Tang G (2016). Investigating the contribution of social genetic effect to longer selection response in a ten generations breeding programme simulate. Ital J Anim Sci..

