# Supplementary material for: Longitudinal analysis of direct and indirect effects on average daily gain in rabbits using a structured antedependence model
Source: Genet Sel Evol. 2018 May 10;50:25. doi: 10.1186/s12711-018-0395-9 (PMC5946580; doi:10.1186/s12711-018-0395-9)
Supplement: Supplementary file 1 — Additional file 1. Detailed description of a SAD1-12 model. [file 12711_2018_395_MOESM1_ESM.docx]

**Additional file 1 : Detailed description of a SAD1-12 model**

A SAD1-12 applied to the permanent effect ***p*** leads to:

$p_{i}\left( w_{1} \right)=e_{p,i}\left( w_{1} \right)$,

$p_{i}\left( w_{2} \right)=\theta_{1,2}p_{\boldsymbol{i}}\left( w_{1} \right){+e}_{p,i}\left( w_{2} \right)$,

$p_{i}\left( w_{j} \right)=\theta_{1,j}p_{\boldsymbol{i}}\left( w_{j-1} \right){+e}_{p,i}\left( w_{j} \right)$,

with $\theta_{1,j}=a_{10}+a_{11}w_{j}$ and $var(e_{p}\left( w_{j} \right))=exp\left( b_{0}+b_{1}w_{j}+b_{2}w_{j}^{2} \right)$.

Thus, ${var\left( p\left( w_{1} \right) \right)=\Sigma}_{p}\left( w_{1},w_{1} \right)=exp\left( b_{0}+b_{1}w_{1}+b_{2}w_{1}^{2} \right)$, ${var\left( p\left( w_{2} \right) \right)=\Sigma}_{p}\left( w_{2},w_{2} \right)=\theta_{1,2}^{2}var(p\left( w_{1} \right))+exp\left( b_{0}+b_{1}w_{2}+b_{2}w_{2}^{2} \right)$ and ${cov\left( p\left( w_{1} \right),p\left( w_{2} \right) \right)=\Sigma}_{p}\left( w_{1},w_{2} \right)=\theta_{1,2}\Sigma_{p}\left( w_{1},w_{1} \right)$. Parameters $a_{10}$, $a_{11}$, $b_{0}$ and $b_{1}$ must be estimated.
