# Supplementary material for: Longitudinal analysis of direct and indirect effects on average daily gain in rabbits using a structured antedependence model
Source: Genet Sel Evol. 2018 May 10;50:25. doi: 10.1186/s12711-018-0395-9 (PMC5946580; doi:10.1186/s12711-018-0395-9)
Supplement: Supplementary file 2 — Additional file 2. Variance components used for the simulation. [file 12711_2018_395_MOESM2_ESM.docx]

**Additional file 2: Variance components used for the simulation**

**Genetic covariance matrix for strong genetic antagonism simulation**

|  |  | Direct | | | | | Indirect | | | | |
| --- | --- | --- | --- | --- | --- | --- | --- | --- | --- | --- | --- |
|  |  | Wk1 | Wk2 | Wk3 | Wk4 | Wk5 | Wk1 | Wk2 | Wk3 | Wk4 | Wk5 |
| Direct | Wk1 | 8.03 |  |  |  |  |  |  |  |  |  |
|  | Wk2 | 7.76 | 13.00 |  |  |  |  |  |  |  |  |
|  | Wk3 | 6.68 | 11.21 | 13.47 |  |  |  |  |  |  |  |
|  | Wk4 | 5.01 | 8.40 | 10.09 | 10.26 |  |  |  |  |  |  |
|  | Wk5 | 3.21 | 5.37 | 6.45 | 6.57 | 6.20 |  |  |  |  |  |
| Indirect | Wk1 | **-0.36** | **-0.35** | **-0.30** | **-0.23** | **-0.15** | 0.44 |  |  |  |  |
|  | Wk2 | **-0.19** | **-0.38** | **-0.32** | **-0.24** | **-0.16** | 0.11 | 0.26 |  |  |  |
|  | Wk3 | **-0.20** | **-0.35** | **-0.39** | **-0.29** | **-0.19** | 0.15 | 0.18 | 0.23 |  |  |
|  | Wk4 | **-0.19** | **-0.32** | **-0.36** | **-0.30** | **-0.19** | 0.14 | 0.16 | 0.20 | 0.22 |  |
|  | Wk5 | **-0.20** | **-0.35** | **-0.39** | **-0.31** | **-0.20** | 0.14 | 0.18 | 0.21 | 0.24 | 0.31 |

To simulate moderate genetic antagonism, covariance components in bold were divided by 2. To simulate weak genetic antagonism, covariance components in bold were divided by 4

**Group covariance matrix**

|  | Wk1 | Wk2 | Wk3 | Wk4 | Wk5 |
| --- | --- | --- | --- | --- | --- |
| Wk1 | 5.60 |  |  |  |  |
| Wk2 | -1.33 | 7.45 |  |  |  |
| Wk3 | 0.35 | -1.94 | 9.60 |  |  |
| Wk4 | -0.10 | 0.55 | -2.71 | 12.35 |  |
| Wk5 | 0.03 | -0.17 | 0.83 | -3.76 | 15.91 |

**Residual covariance matrix**

|  | Wk1 | Wk2 | Wk3 | Wk4 | Wk5 |
| --- | --- | --- | --- | --- | --- |
| Wk1 | 23.06 |  |  |  |  |
| Wk2 | -0.57 | 28.12 |  |  |  |
| Wk3 | 0.05 | -2.38 | 34.46 |  |  |
| Wk4 | -0.01 | 0.34 | -4.97 | 42.48 |  |
| Wk5 | 0.00 | -0.07 | 1.01 | -8.66 | 52.67 |
