# Supplementary material for: Longitudinal analysis of direct and indirect effects on average daily gain in rabbits using a structured antedependence model
Source: Genet Sel Evol. 2018 May 10;50:25. doi: 10.1186/s12711-018-0395-9 (PMC5946580; doi:10.1186/s12711-018-0395-9)
Supplement: Supplementary file 3 — Additional file 3. Correlation matrix among (co)variance component estimates. [file 12711_2018_395_MOESM3_ESM.docx]

**Additional file 3: Correlation matrix among (co)variance component estimates**

Close inspection of the correlation between estimates at the level of the (co)variance components is used to evaluate if the variance component of the different random effects are properly disentangle when estimated by a SAD model.
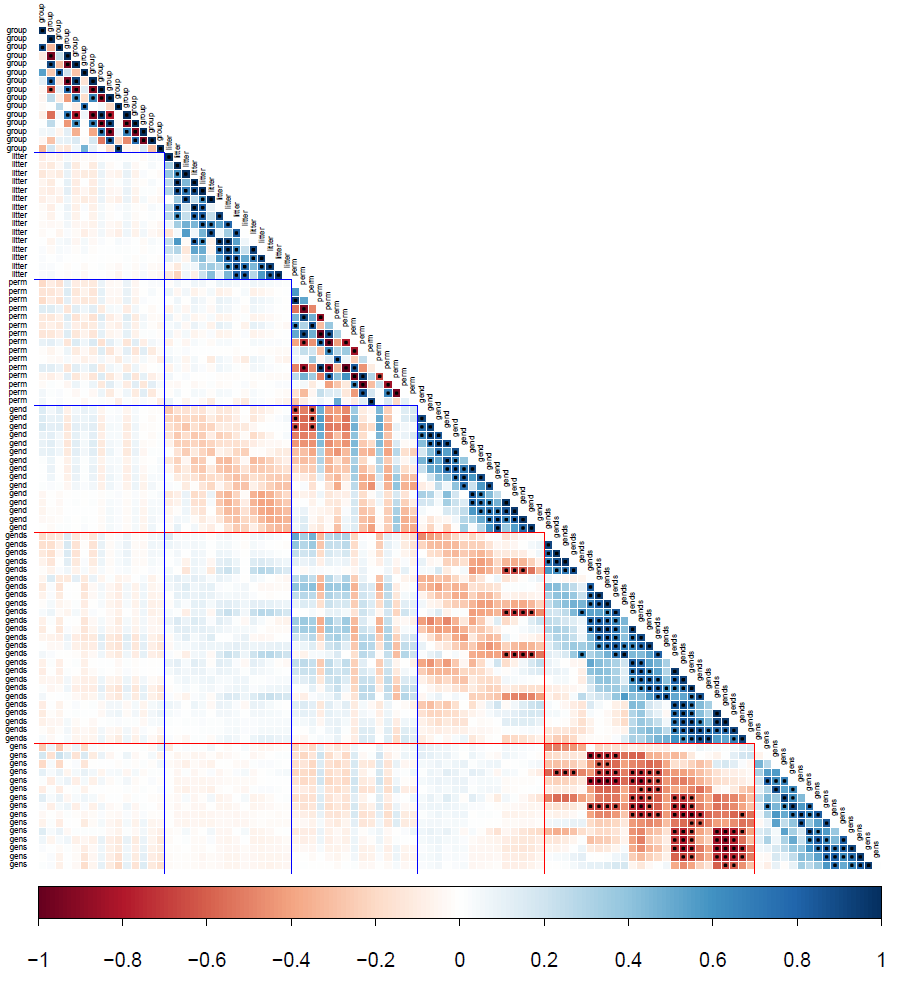


For each random effect as well as for the covariances between direct and social genetic effects (gends), components are presented in lower triangular matrix order. “gend”, “gens”, “group”, “litter” and “perm” corresponds to direct, social genetic, group, litter and pseudo-permanent effects in eq1, respectively.
